# Epstein-Barr virus oncoprotein LMP1 mediates survivin upregulation by p53 contributing to G1/S cell cycle progression in nasopharyngeal carcinoma

**DOI:** 10.3892/ijmm.2012.889

**Published:** 2012-01-17

**Authors:** LILI GUO, MIN TANG, LIFANG YANG, LANBO XIAO, ANN M. BODE, LILI LI, ZIGANG DONG, YA CAO

**Affiliations:** 1 Cancer Research Institute, Xiangya School of Medicine, Central South University, Changsha, Hunan 410078; 2 Tianjin Key Laboratory of Lung Cancer Metastasis and Tumor Microenvironment, Tianjin Lung Cancer Institute, Tianjin Medical University General Hospital, Tianjin 300052; 3 Carcinogenesis and Invasion Key Laboratory of Education Ministry of China, Central South University, Changsha 410078, P.R. China; 4 The Hormel Institute, University of Minnesota, Austin, MN 55912, USA

**Keywords:** Epstein-Barr virus, latent membrane protein 1, p53, survivin, cell cycle

## Abstract

Latent membrane protein 1 (LMP1) is an important oncogenic protein encoded by Epstein-Barr virus (EBV) and plays an important role in the development of nasopharyngeal carcinoma (NPC). Our previous study has shown that p53 protein was accumulated and phosphorylated in NPC, implying its transcription factor activity in NPC tumorigenesis. However, the biological function and potential downstream target of p53 mediated by LMP1 in NPC remain unknown. In this study, we found that LMP1 simultaneously induced upregulation of both p53 and survivin at the protein level, as well as their phosphorylation. Knockdown of p53 by siRNA revealed that LMP1 increased survivin expression by p53 directly. Furthermore, we found that LMP1 upregulated survivin by p53 at the transcriptional level by increasing p53-mediated *survivin* promoter activity and DNA binding activity. Moreover, LMP1 induced the co-localization of p53 and survivin in the nucleus, conferring to their related functions in NPC tumorigenesis. We further found that p53 promoted G1/S cell cycle progression, but did not induce apoptosis in LMP1-positive NPC cells. Collectively, these findings suggest that p53 acting as a transcription factor promotes the transcriptional activity of *survivin*, and further increases its protein expression and phosphorylation in the regulation of LMP1, thus, leading to G1/S cell cycle progression with no effect on apoptosis in NPC tumorigenesis.

## Introduction

Epstein-Barr virus (EBV) has been identified as a DNA virus that is associated with human malignancies, including Burkitt’s lymphoma, Hodgkin’s disease and nasopharyngeal carcinoma (NPC) (1,2). Latent membrane protein 1 (LMP1) is an important oncogenic protein among EBV-encoded proteins and is expressed in up to 90% of NPC patients. It thus plays an important role in the development of NPC (3,4). Previous work has shown that LMP1 is involved in multiple NPC biological processes including cell proliferation, apoptosis, invasion and metastasis, by inducing the activation of NF-κB, JAK/STAT, PKC and the MAPK signaling pathways (5) and their downstream genes including *p53* and *survivin* (6,7).

The tumor suppressor gene *p53* is a critical mediator of cell cycle, DNA repair, cell differentiation and apoptosis. Many human tumors are associated with p53 mutations, supporting its pivotal role as a key tumor suppressor in tumorigenesis. Unlike in most human tumors, p53 accumulates in NPC and the mutation rate of p53 is <10% (8,9). Immunohistochemical analysis of NPC biopsies indicates that p53 accumulation is significantly associated with LMP1 expression (10,11). In our previous study, LMP1 was found to increase the transcriptional activity and expression of both wild-type and mutant p53 in NPC (6,12). LMP1 could mediate p53 phosphorylation at Ser15, Ser20, Ser392 and Thr81, indicating potential functional activity of p53 in NPC progression (13). Although other reports have also shown a possible role of p53 in NPC (14,15), the biological function and potential downstream target of accumulated p53 in NPC still remains unclear.

Survivin, a member of the inhibitor of apoptosis (IAP) family identified in 1997 (16), is widely expressed in fetal tissues and most tumor tissues. Survivin is overexpressed in multiple tumors, and corresponds with poor prognosis (17). Survivin is highly expressed at the G2/M phase in a cell cycle-regulated manner, and also promotes G1/S cell cycle progression by translocating into the nucleus and forming a complex with CDK4 (18). As a mitotic substrate of Cdc2/cyclin B1, survivin can be phosphorylated on Thr34 on the mitotic apparatus, which contributes to the regulation of cell division through an interaction with caspase-9 (19). Thus, survivin has dual functions in cell cycle regulation and apoptosis inhibition. We previously showed that LMP1 could increase the activity of survivin through the NF-κB and AP-1 signaling pathways in NPC (7,19). Remarkably, the *survivin* promoter contains a p53 binding element located in the *survivin* 230-bp basic core promoter region (20,21), indicating the possible regulation of survivin by LMP1 via p53 in NPC.

Here, using siRNA technology to knockdown the expression of p53, we found that LMP1 upregulated survivin protein expression and its phosphorylation by p53 due to the transactivation of the *survivin* promoter. LMP1 caused the translocation of p53 into the nucleus with survivin, suggesting that survivin is the key downstream target of p53. We further found that accumulated p53 by LMP1 promoted G1/S cell cycle progression, but did not induce apoptosis in NPC pathogenesis.

## Materials and methods

### Cell culture and plasmids

CNE1 cells comprise an LMP1-negative, highly differentiated nasopharyngeal carcinoma cell line with mutant p53 (8). CNE1-LMP1 NPC cells are a stably transfected cell line, which was established by introducing *LMP1* cDNA into CNE1 NPC cells. CNE1 and CNE1-LMP1 NPC cells were maintained in RPMI-1640 medium supplemented with 10% fetal calf serum. NP69-pLNSX and NP69-LMP1 cells are SV40-transformed, immortalized normal nasopharyngeal cell lines (33), with no p53 mutation (12). These cells were cultured in defined keratinocyte serum-free medium (KSFM) (Gibco Life Technologies, Basel, Switzerland) supplemented with growth factors. All cell lines were maintained at 37°C with 5% CO_2_. The pSUPER-p53siRNA is a plasmid with an siRNA targeting p53 inserted in the pSUPER vector. This vector was a generous gift from Professor Qiao Wu (Key Laboratory of Ministry of Education for Cell Biology and Tumor Cell Engineering, Xiamen University, China). The pGL3-Sur1.8kb (pGL3.basic.survivin.promoter1.8kb) is a *survivin* promoter-luciferase reporter construct obtained from Professor Ningzhi Xu (Cancer Research Institute, Chinese Academy of Medical Sciences). The pSV-β-galactosidase control vector was purchased from Promega (Southampton, UK).

### Western blot analyses

Cells were collected and washed with ice-cold PBS 3 times. Lysis buffer (50 mM Tris-HCl, 1 mM EDTA, 20 g/l SDS, 5 mM DTT and 10 mM PMSF) was added and cells were left on ice for 30 min. Cells were then boiled for 10 min followed by ultrasonication for 30 sec. All procedures were carried out at 4°C. Proteins were collected by centrifugation at 10,000 × g for 10 min. Protein concentrations were determined using the BCA protein assay reagent (Pierce Chemical Co., Rockford, IL), with bovine serum albumin as a standard. For western blot analysis, 50 μg of total protein was loaded onto an 8–12% Tris-glycine polyacrylamide gel and subjected to electrophoresis. Proteins were visualized by ECL chemiluminescence reagents (Pierce Chemical Co.). Primary antibodies specific for human p53 (sc-126), phosphorylated p53 (Ser20) (sc-18079), survivin (sc-8807), phosphorylated survivin (Thr34) (sc-23758), caspase-3 (sc-7272), CDK2 (sc-163), CDK4 (sc-260), cyclin D1 (sc-20044), α-tubulin (sc-5286), nucleolin (sc-8031) and secondary antibodies for goat anti-rabbit IgG-HRP (sc-2004) and goat anti-mouse IgG-HRP (sc-2005) were all purchased from Santa Cruz Biotechnology, Inc. (Santa Cruz, CA).

### Quantitative real-time PCR

Total-RNA was isolated using the TRIzol (Invitrogen, Carlsbad, CA, USA) reagent following the manufacturer’s suggested protocols. Reverse transcription PCR was performed by using the Reverse Transcription System (Promega, Madison, WI). Each 25 μl of PCR reaction mixture was prepared using the SYBR^®^ Premix Ex Taq™ kit (Takara Bio, Inc.). The primer sequences for *survivin* were as follows: forward, 5′-AGGTGCCTGTTGAATCTG-3′ and reverse, 5′-GACGCTTCCTATCACTCTATT-3′; the *β-actin* primer sequences were forward, 5′-TTCCAGCCTTCCTTCCTGGG-3′ and reverse, 5′-TTGCGCTCAGGAGGAGCAAT-3′. The amplification conditions were set up according to the protocol included with the SYBR Premix Ex Taq™ kit. Samples were run in triplicate for each experiment using the 7500 Real-Time PCR System (Applied Biosystems, Foster City, CA). The relative amount of mRNA was calculated using the comparative CT method after normalization to *β-actin* mRNA levels.

### Luciferase assays

*Survivin* promoter-luciferase reporter constructs (pGL3-Sur1.8kb) in combination with the pSV-β-galactosidase control construct were transfected into cells 48 h after pSUPER-p53-siRNA transfection, using Lipofectamine™ 2000, according to protocols provided by the manufacturer (Invitrogen). After 24 h, cells were harvested and lysed with reporter lysis buffer (RLB; Promega) and the luciferase activity was determined using the Luciferase Reporter Assay System (Promega) according to the manufacturer’s instructions. Experiments were performed in quadruplicate, and the statistical significance was assessed by a paired t-test.

### Electrophoretic gel mobility shift assay (EMSA)

Nuclear extracts were prepared by the use of the NE-PER Nuclear and Cytoplasmic Extraction kit (Pierce) in accordance with the manufacturer’s protocol. The p53 oligonucleotide probes were synthesized (Invitrogen). The sequences were as follows: (Bio)-p53, sense, 5′-(Biotin)-GCCTAAGAGGGCGTGCGCTC CCGACATGCCCCGCGG-3′ and antisense, 5′-(Biotin)-CC GCGGGGCATGTCGGGAGCGCACGCCCTCTTAGGC-3′; NS-p53, sense, 5′-CAGGGACGATATGGATAGATTTC GCTGGGT-3′ and antisense, 5′-ACCCAGCGAAATCTATCC ATATCGTCCCTG-3′; (Bio)-mut-p53, sense, 5′-(Biotin)-GC CTAAGAGGTCTCTCGCTCCCGAAAGACCCCGCGG-3′ and antisense, 5′-Biotin-CCGCGGGGTCTTTCGGGAGC GAGAGACCTCTTAGGC-3′. EMSAs were performed using protocols provided in the LightShift™ Chemiluminescent EMSA kit (Pierce). Labeled (2 μl) oligonucleotides and nucleoproteins (10 μg) were mixed in binding buffer (Pierce) and incubated for 15 min at room temperature. Samples were subjected to electrophoresis in 5% non-denaturing polyacrylamide gel and transferred to a Biodyne™ B Nylon membrane (Pierce). The protein bands were detected using ECL chemiluminescence reagents (Pierce).

### Chromatin immunoprecipitation (ChIP) assay

ChIP assays were performed as described previously (21). Briefly, chromatin was incubated overnight with p53 antibody, or no antibody added as a negative control. The sequences of the primers were *survivin-*F, 5′-TGGGTGCCCCGACGT-3′, and *survivin*-R, 5′-GAAGGGCCAGTTCTTGAATGTAGA-3′.

### Immunofluorescence analysis

Cells were cultured in 6-well plates and then washed with cold phosphate-buffered saline (PBS) and fixed with cold 3.7% polyformaldehyde for 30 min. The primary antibodies were diluted 1:200 in PBS and incubated with the cells at 4°C overnight followed by washing with 0.25% PBS-Triton X-100. Fluorescein-labeled IgG was diluted 1:1,000 with PBS and incubated with the cells to bind with the primary antibodies, anti-mouse IgG labeled with Cy3 (Sigma Chemical Co., St. Louis, MO, USA) for p53, anti-rabbit IgG labeled with FITC (Sino-American Biotechnology, Shanghai, China) for survivin, and Hochest 33258 to stain nuclei. Cellular localization of proteins was observed under a fluorescence microscope or by Laser Scanning Confocal Microscopy (Leica Microsystems Inc., USA).

### Apoptosis and cell cycle analyses by FCM

Cultured cells were harvested and washed with 1X PBS and then suspended in 1X PBS containing 0.1% glucose. Cold 70% ethanol was added and mixed immediately and then the ethanol was removed by centrifugation at 1,000 rpm. Cells were washed 2 times with PBS, 100 μl of PC buffer were added and the solution was incubated at room temperature for 30 min. The cells were suspended in 100 μl PBS, 10 mg/ml RNase (10 μl), and propidium iodide solution (10 μl) and then incubated at room temperature for 30 min. Cells were transferred to FACS tubes and analyzed by flow cytometry.

### Statistical analysis

Data are expressed as means ± SD and statistical comparisons were performed using the Student’s t-test. A value of P<0.05 was considered statistically significant.

## Results

### LMP1 upregulated survivin expression and its Thr34 phosphorylation by p53

**Figure 1:**
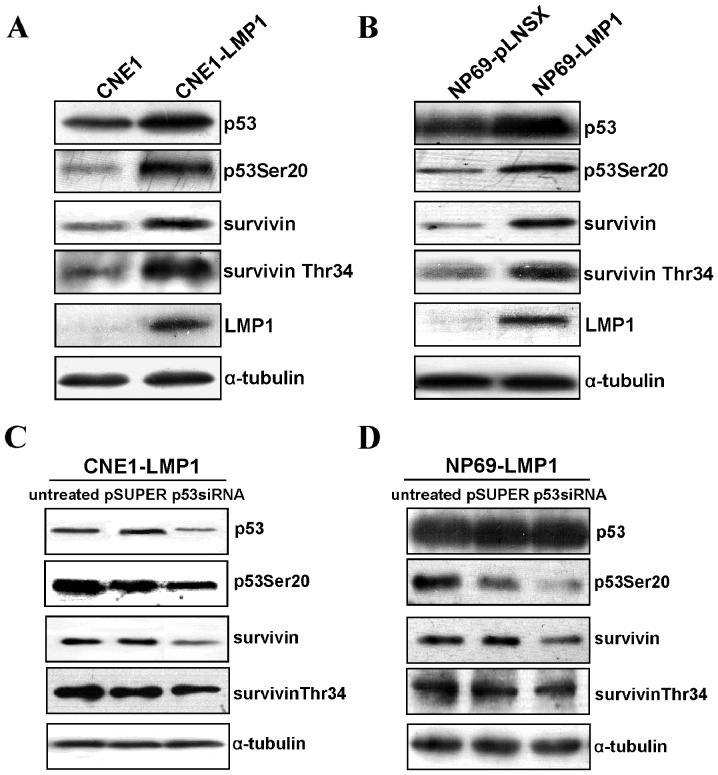
LMP1 upregulates survivin expression and its Thr34 phosphorylation by p53 (A and B) in CNE1 and NP69 cells expressing LMP1 and (C and D) using siRNA targeting p53. α-tubulin was used as an internal control. Cells transfected with siRNA targeting p53 were incubated for 72 h. Expression of p53, phosphorylated p53 (Ser20), survivin, and phosphorylated survivin (Thr34) was detected. Untransfected cells (untreated) and cells transfected with pSUPER constructs were used as controls.

We previously found that LMP1 could upregulate the expression and activities of both p53 and survivin. We next investigated the correlation of increased survivin and p53 mediated by LMP1. Phosphorylated p53 (Ser20) and survivin (Thr34) were selected to reflect p53 and survivin activity, respectively (9,16). We found that LMP1 induced the expression of p53 and survivin simultaneously in the LMP1-positive CNE1 and NP69 cell lines, as well as the phosphorylation of p53 (Ser20) and survivin (Thr34) (Fig. 1A and B). We further investigated their correlation using a loss-of-function assay. Knockdown of p53 by siRNA showed that survivin protein level and phosphorylated survivin (Thr34) were decreased in p53-depleted CNE1-LMP1 and NP69-LMP1 cells (Fig. 1C and D). These data suggest that LMP1 increases survivin expression and activity by p53 in NPC.

### LMP1 increases survivin expression by p53 at the transcriptional level

**Figure 2:**
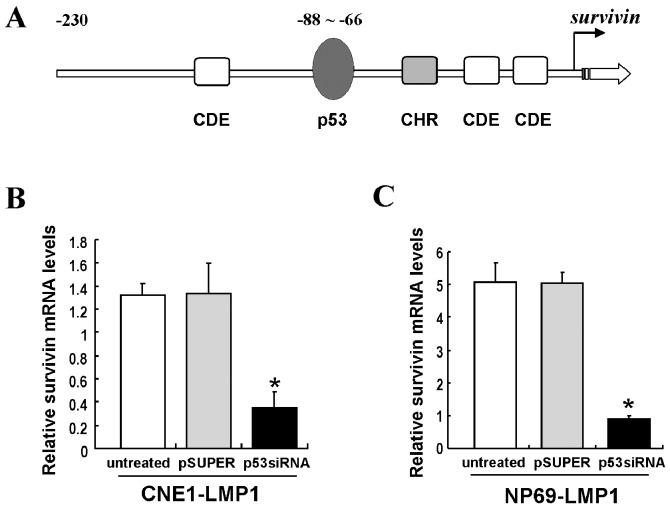
(A) Sequence of the *survivin* promoter in the minimal p53-binding region. The arrow indicates the start site of *survivin* transcription. (B and C) Q-PCR showed that LMP1 reduced the expression of *survivin* mRNA in p53siRNA-transfected CNE1-LMP1 and NP69-LMP1 cell lines (^*^P<0.05).

**Figure 3:**
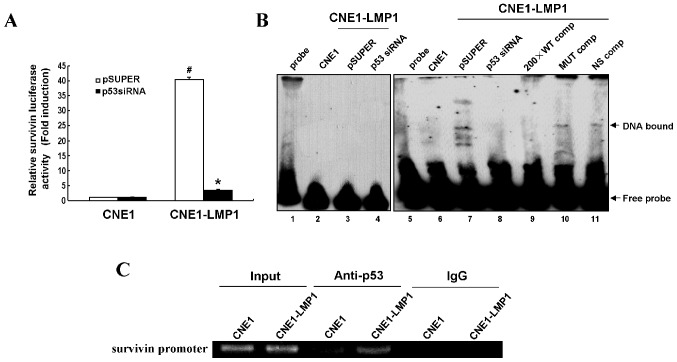
Transcriptional upregulation of *survivin* by p53 is induced by LMP1. (A) LMP1 increased survivin promoter activity (^#^P<0.05) and survivin promoter activity was significantly decreased in the p53 siRNA-transfected CNE1-LMP1 cell line (^*^P<0.05). The relative luciferase activity was normalized to the value of pRL-SV40 luciferase activity. Results are expressed as the fold induction of *survivin* promoter activity, CNE1 cells transfected with pSUPER constructs, which was assigned a value of 1. (B) LMP1 induces p53-DNA binding activity as measured by EMSA. A biotin-labeled wild-type p53 oligonucleotide probe was incubated with nuclear extracts of CNE1, pSUPER constructs and p53 siRNA-transfected CNE1-LMP1 NPC cells (lanes 6–8) in the presence of a 200-fold excess of unlabeled wild-type p53 oligonucleotide (lane 9), a 200-fold excess of an unlabeled oligonucleotide containing a 7 bp mutation of the p53 sequence (designated mut, lane 10), or a 200-fold excess of non-specific unlabeled oligonucleotide (NS, lane 11). A biotin-labeled mutant p53 oligonucleotide probe was used as a negative control (lanes 1–4). (C) ChIP assay to show that p53 binds to survivin promoter in CNE1-LMP1 cells.

The *survivin* promoter contains a p53 binding element (Fig. 2A). We thus further investigated whether LMP1 increased survivin expression by p53 at the transcriptional level. Quantitative PCR (Q-PCR) showed that *survivin* mRNA levels were significantly decreased in p53-siRNA-transfected CNE1-LMP1 and NP69-LMP1 cells (P<0.05) (Fig. 2B and C). The *survivin* promoter-luciferase reporter (pGL3-Sur1.8kb) was used to assess the effect of *survivin* promoter activity by p53 in the regulation of LMP1. Results revealed that the *survivin* promoter activity was significantly increased in CNE1-LMP1 cells compared with CNE1 cells (P<0.05), while a dramatic inhibition of the *survivin* promoter activity appeared in p53-depleted CNE1-LMP1 cells but not in CNE1 cells (P<0.05) (Fig. 3A).

Next, EMSA was performed to verify whether LMP1 promoted p53 binding to the *survivin* promoter. Biotin-labeled wild-type or mutant p53 oligonucleotide probes were incubated with nuclear extracts of CNE1, CNE1-LMP1 or p53-depleted CNE1-LMP1 cells. Our data show that LMP1 obviously inhibited p53-survivin DNA binding activity (Fig. 3B, lanes 6–8). A biotin-labeled mutant p53 oligonucleotide probe was used as a negative control (Fig. 3B, lanes 1–4). A 200-fold excess of an unlabeled wild-type p53 oligonucleotide probe efficiently inhibited the p53-DNA binding activity (Fig. 3B, lane 9), whereas an unlabeled mutant p53 probe (Fig. 3B, lane 10) or a non-specific unlabeled p53 probe (Fig. 3B, lane 11) could not, confirming the binding specificity of these up-shifts. ChIP assay further demonstrated increased binding of p53 to the *survivin* promoter region mediated by LMP1 *in vivo* (Fig. 3C). Thus, survivin may act as a transcription target of p53 in the regulation of LMP1, responsible for its upregulation in NPC.

### LMP1 induces the co-localization of p53 and survivin in the nucleus

**Figure 4:**
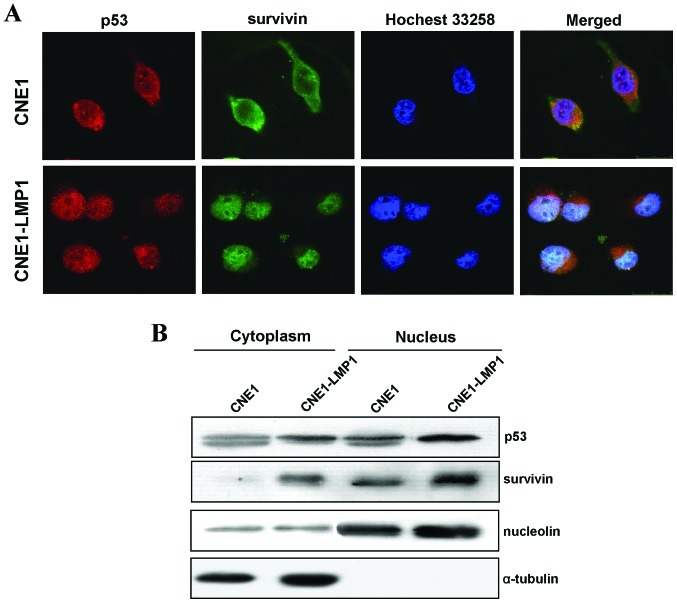
(A) LMP1 promotes p53 and survivin nuclear accumulation, analyzed by immunofluorescence. Cy3 (red) staining indicates p53 localization, FITC (green) staining indicates survivin localization, and Hochest 33258 (blue) indicates nuclear staining. (B) Nuclear and cytoplasmic proteins were collected from CNE1 and CNE1-LMP1 cells and then separated by western blot analysis to detect survivin and p53. α-tubulin was used as a marker for cytoplasmic proteins and nucleolin for nuclear proteins.

Both functional p53 and survivin are located in the nucleus. Therefore, an immunofluorescence assay was used to examine their cellular localization. We observed that survivin and p53 were translocated into the nucleus of CNE1-LMP1 cells cooperatively (Fig. 4A). Western blot analyses of cellular fractions showed that both p53 and survivin were detected in the cytoplasm factions in CNE and CNE1-LMP1 cells, but LMP1 obviously increased the expression of p53 and survivin in the nucleus (Fig. 4B), confirming the immunofluorescence results. Thus, LMP1 can promote the nuclear accumulation of p53 and survivin, facilitating their functional execution in NPC tumorigenesis.

### Activation of p53 signaling by LMP1 results in G1/S cell cycle progression but does not induce apoptosis

**Figure 5:**
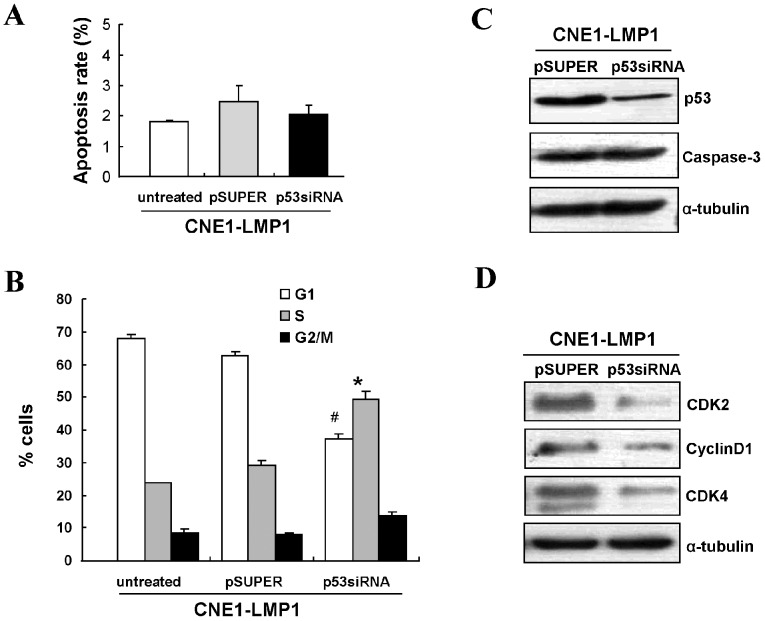
Apoptosis and cell cycle analysis were examined by FCM and western blot analysis. (A) FCM results show that the apoptosis rate is less changed after p53 gene knockdown in CNE1-LMP1 cells, with P>0.05 by the Student’s t-test, compared with the controls. (B) Western blot analysis shows that the caspase-3 protein level does not change in CNE1- LMP1 cells transfected with p53-siRNA. (C) FCM results show that targeting p53 by siRNA could increase the number of CNE1-LMP1 cells in the S phase (^*^P<0.05), but decrease those in the G0/G1 phase (^#^P<0.05). The number of cells in the G2/M phase remained unchanged in p53siRNA transfected cells. (D) Downregulated G1/S phase checkpoint-related proteins, CDK2, CDK4 and cyclin D1, were observed in CNE-LMP1 cells transfected with p53siRNA.

As survivin possesses a dual function in promoting cell cycle progression and inhibiting apoptosis, and p53 is responsible for the activation of survivin, we next investigated the effects of p53 on cell cycle and apoptosis mediated by LMP1 using siRNA to knockdown p53 expression. Flow cytometry showed that knowdown of p53 minimally affected the apoptosis rate of LMP1-positive cells (Fig. 5A). Consistent with this result, no difference in caspase-3 protein levels was observed in either of these two groups (Fig. 5B).

Cell cycle analyses demonstrated that targeting p53 by siRNA could increase the number of LMP1-positive cells in the S phase (P<0.05) and decrease those in G0/G1 (P<0.05) (Fig. 5C). Western blot analysis further confirmed that LMP1 increased the expression of G1/S checkpoint related proteins, CDK2, CDK4 and cyclin D1 (Fig. 5D). These data suggest that activated p53 signaling by LMP1 promotes G1/S cell cycle progression, but not apoptosis in NPC tumorigenesis.

## Discussion

p53 is well known as a tumor suppressor gene, and its mutation has been found to be the most frequent genetic alteration in human malignancy. However, p53 overexpression or accumulation with a rare mutation has been identified in NPC, unlike other types of cancer. More and more evidence demonstrates that p53 overexpression occurs at an early stage in the development of NPC (22) and is associated with an advanced disease stage with a poor prognosis (23). We and others have shown that p53 can be phosphorylated and activated by LMP1, thus it might be a transcription factor in NPC (12–15). In this study, we further studied the potential downstream target and biological function of p53 mediated by LMP1 in NPC pathogenesis.

Survivin is a central player in regulating cell cycle progression and apoptosis inhibition (24), and the regulation of *survivin* by p53 is complicated. For examples, the p53 transcription factor directly binds the *survivin* promoter alone or in combination with other protein(s), such as E2F, Sin3 or HDAC, leading to the suppression of *survivin* (25,26). The p53 protein regulates survivin phosphorylation by binding to the subunit of Cdc2/cyclin B1 kinase (19), conversely, survivin could regulate p53 through caspase/Mdm2 (27) and Aurora B (28). Moreover, mutated p53 can stimulate the expression of survivin through one or more signaling pathways (29). By knockdown of p53 protein expression with siRNA, we found that LMP1 promotes p53-mediated survivin upregulation by increasing *survivin* promoter activity and p53-survivin DNA binding activity, suggesting the complexity of the regulation of p53 on survivin mediated by viral oncoprotein LMP1 in NPC.

The nuclear localization is critical to the transcriptional activity of p53 and the antiapoptotic function of survivin. Recently, survivin was reported to be preferentially degraded in the nucleus in the G1 phase mediated by Cdh1 (30), and forced expression of survivin in the nucleus is sufficient to inhibit apoptosis in human cells. Interestingly, a recent research report indicated that overexpression of survivin in the nucleus could increase control over the G1/S checkpoint by increasing the nuclear accumulation of cyclin D1 and CDK4, following pRb phosphorylation, which enhanced viral protein expression and viral replication (31). In NPC, the expression of survivin in the nucleus is associated with poor prognosis (23). Our previous data showed that LMP1 could induce the expression of survivin and CDK4 simultaneously and promote their co-localization in the nucleus, contributing to G1/S cell cycle progression in NPC (32). Here, we observed that LMP1 increased nuclear localization of both p53 and survivin, required for their function execution in NPC progression. Activated p53 signaling by LMP1 mainly promoted G1/S cell cycle progression but did not induce apoptosis in NPC cells, consistent with our previous findings.

**Figure 6:**
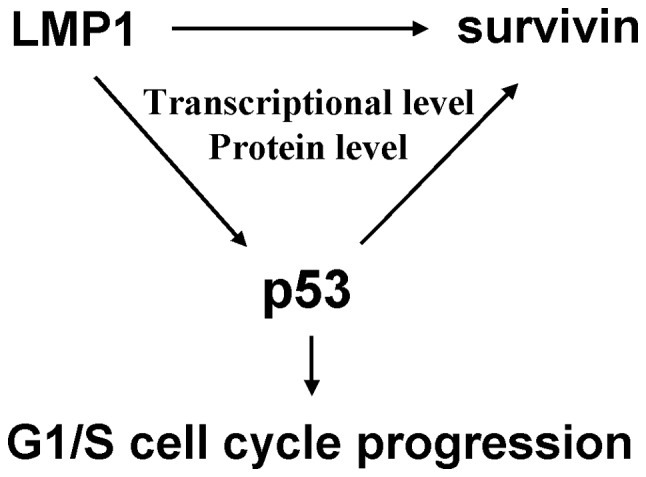
Proposed model of p53-induced G1/S cell cycle progression by upregulating survivin expression mediated by LMP1 in NPC pathogenesis.

In summary, our study verified that p53 as a transcriptional factor, could upregulate survivin expression in both the transcriptional level and protein level mediated by LMP1, and further stabilized the nuclear localization of survivin, ultimately resulting in G1/S cell cycle progression but not the induction of apoptosis in NPC (Fig. 6). These results extend our knowledge of the functional activity and molecular mechanism of accumulated p53 in NPC pathogenesis.
